# Addressing sexual violence: A FIGO Committee on Women Facing Crises consensus statement

**DOI:** 10.1002/ijgo.71177

**Published:** 2026-07-07

**Authors:** Kristina Jariene, Margit Endler, Rubina Sohail, Lubna Hassan, Algirdas Jaras, Atziri Ramirez‐Negrin

**Affiliations:** ^1^ Department of Obstetrics and Gynecology Medical Academy, Lithuanian University of Health Sciences Kaunas Lithuania; ^2^ FIGO Committee on Women Facing Crises, FIGO London UK; ^3^ Department of Women's and Children's Health Karolinska Institutet Stockholm Sweden; ^4^ School of Public Health University of Cape Town Cape Town South Africa; ^5^ Department of Obstetrics and Gynecology Hameed Latif Hospital Affiliate of Rashid Latif Medical University Lahore Pakistan; ^6^ Department of Obstetrics and Gynaecology The Woman's Hospital Peshawar Pakistan; ^7^ Department of Psychiatry Medical Academy, Lithuanian University of Health Sciences Kaunas Lithuania; ^8^ Department of Urogynecology Hospital Dr Manuel Gea Gonzalez Mexico City Mexico

**Keywords:** gender‐based violence, integrated care models, legal reform, multisectoral collaboration, NGOs and advocacy, psychosocial support, public awareness campaigns, sexual violence, survivor‐centered care, trauma‐informed health care

## Abstract

Sexual violence (SV) is a global crisis that affects individuals across all demographics, with disproportionate impact on women, children, and marginalized communities. This paper explores the prevalence, consequences, and barriers survivors face in accessing care and justice. It highlights the physical, psychological, and social toll of SV against women and emphasizes the importance of survivor‐centered approaches in healthcare, legal, and social support systems. In this paper, the FIGO Committee on Women Facing Crises outlines key recommendations for governmental authorities, healthcare providers, and non‐governmental organizations (NGOs) for comprehensive care of SV survivors, including the establishment of integrated care models, legal reforms, and public awareness campaigns. Successful examples are presented as scalable solutions. This paper concludes by advocating for collaborative, multisectoral efforts and long‐term strategies between relevant stakeholders to meet the needs of SV survivors with dignity and justice.

## 1 INTRODUCTION

Sexual violence remains one of the most pervasive violations of human rights worldwide and represents a major global public health concern. Survivors frequently face profound physical, psychological, and social consequences, while barriers within health, legal, and social systems often limit access to timely and respectful care. This paper presents the consensus recommendations of the International Federation of Gynecology and Obstetrics (FIGO) Committee on Women Facing Crises, aimed at strengthening survivor‐centered, multisectoral responses to sexual violence globally.

The recommendations presented in this paper were developed by the FIGO Committee on Women Facing Crises, an international expert group composed of clinicians and researchers with expertise in sexual and gender‐based violence, reproductive health, and crisis settings.

This consensus statement was informed by a narrative review of the existing literature and international guidelines related to sexual violence, survivor‐centered care, and multisectoral response frameworks.

Relevant international guidelines, including publications from WHO, United Nations (UN) agencies, and peer‐reviewed scientific literature, were reviewed to inform the development of the recommendations. Committee members also contributed regional perspectives from diverse health systems and sociocultural contexts.

Draft recommendations were developed through iterative discussion and expert consultation among committee members. The final recommendations reflect the consensus of the committee and aim to provide practical guidance for governments, healthcare providers, and civil society organizations.

## 2 THE GLOBAL PREVALENCE AND IMPACT OF SEXUAL VIOLENCE

Sexual violence is one of the most widespread violations of human rights worldwide. It disproportionately affects women and girls but also impacts men, boys, and gender‐diverse individuals. A 2018 analysis conducted by WHO on behalf of the UN Interagency Working Group on Violence Against Women revealed staggering statistics: nearly one in three women globally—approximately 30%—has been subjected to physical and/or sexual violence by an intimate partner, a non‐partner, or both during their lifetime.^1^ This figure reflects a pervasive crisis that spans cultural, economic, and geographic boundaries, highlighting the ubiquity of this issue. Estimating changes in the prevalence of sexual violence over time remains challenging due to underreporting, differences in survey methodologies, and evolving definitions of violence. However, available evidence suggests that social instability, armed conflict, forced migration, and economic crises may increase vulnerability to sexual violence in many settings.

At least 120 million girls under the age of 20 years, roughly one in 10, have been forced to engage in sexual acts, although the true prevalence is likely higher due to underreporting. Particularly harrowing is the revelation that 90% of adolescent girls and women who reported forced sexual acts identified their first perpetrator as someone they knew, often a boyfriend or husband.^2^ UNICEF further estimates that 650 million women and girls alive today were subjected to sexual violence during childhood globally, encompassing a broader range of abuse beyond forced sexual acts.^3^ A study conducted in the European Union, which surveyed over 42 000 women across 28 member states, suggests that one in 10 women has experienced sexual violence since the age of 15 years, and one in 20 has been raped. The study further revealed that over 20% of women had endured physical or sexual violence from a current or previous partner, while 10% disclosed experiencing sexual violence as children, perpetuating cycles of trauma and vulnerability.^4^ In Latin America, this number is even higher, with physical and/or sexual violence present in 25% of the female population.^5^ In Africa, the rates are even more alarming: in Eastern and Southern Africa, approximately 42% of women have experienced either physical or sexual intimate partner violence in their lifetime, and approximately 33% of women across the continent have faced some form of physical or sexual violence, underscoring the magnitude of the crisis.^6^, ^7^ The risks are even more severe for migrant women: data indicate that 80% have been raped by the time they reach the USA.^8^

In situations of armed conflict, forced displacement, and humanitarian crises, the risk of sexual violence often increases substantially. Sexual violence has been widely documented as a weapon of war and a tactic used to terrorize communities and destabilize civilian populations. At the same time, humanitarian emergencies can weaken legal protections and health systems, leaving survivors with limited access to medical care, psychosocial support, and justice. Strengthening survivor‐centered health services and protection mechanisms in crisis and displacement settings is therefore a critical component of global responses to sexual violence.

## 3 THE PHYSICAL, PSYCHOLOGICAL, AND SOCIAL TOLL

The consequences of sexual violence are far‐reaching, affecting survivors' physical, psychological, and social well‐being. Survivors face a heightened risk of contracting HIV and other sexually transmitted infections, often due to forced, unprotected intercourse. The physical repercussions extend to unwanted pregnancies, chronic pain, and other medical complications.^9^, ^10^ Psychologically, survivors often experience profound trauma, manifesting as anxiety, depression, post‐traumatic stress disorder (PTSD), and even suicide. The lifetime prevalence of PTSD for women who have been sexually assaulted is 50%. Moreover, sexual assault is the most frequent cause of PTSD in women, with one study reporting that 94% of women experienced PTSD symptoms during the first 2 weeks after an assault.^10^, ^11^, ^12^ Studies have linked sexual violence to increased risk of substance abuse as survivors attempt to cope with their ordeal. For child survivors, the impacts are especially devastating. Sexual violence during formative years can impair a child's ability to develop healthy relationships, care for themselves, or achieve emotional stability, perpetuating cycles of vulnerability into adulthood. Social isolation is another common consequence. Survivors frequently face rejection by their families and communities, particularly in conservative or patriarchal societies where victim‐blaming is prevalent. This rejection often leads to economic insecurity, spousal abandonment, and reduced access to social support systems, compounding the trauma.^2^, ^10^, ^13^, ^14^, ^15^, ^16^

## 4 A SILENT EPIDEMIC: UNDERREPORTING AND ITS IMPLICATIONS

Sexual violence remains one of the most underreported crimes worldwide. Despite its widespread occurrence, most victims never tell anyone. Only 14% of women who experience intimate partner violence report the incident to authorities, and just 13% report non‐partner sexual violence.^4^, ^17^ Among college‐aged women, nearly 80% of student survivors choose not to report sexual violence, and 12% say they did not consider the incident to be serious enough.^18^ The reasons for this silence are multifaceted and ingrained in societal, psychological, and systemic barriers. Survivors often fear being judged, disbelieved, or blamed for the incident. For many, the stigma attached to sexual violence compounds the trauma, discouraging them from seeking medical and legal assistance.

The situation is even more complex in LGBTQ+ communities. Fear of being outed, discrimination, shame, victim‐blaming, and previous experiences of exclusion may create additional barriers to disclosure and help‐seeking. Among LGBTQ+ post‐secondary students, non‐reporting of different forms of sexual violence was very high, ranging from approximately 87% to 92%, depending on the type of incident. Lack of confidence in institutional responses was also common, with 57%–75% of non‐reporting survivors indicating that they did not believe any action would be taken. These barriers reinforce a cycle of silence, mistrust, and invisibility.^18^, ^19^, ^20^, ^21^

Furthermore, healthcare systems are not immune to challenges. Emergency departments and physicians often fail to identify a history of sexual violence unless explicitly disclosed by survivors. This failure is compounded when injuries resulting from violence are not documented, depriving survivors of crucial evidence for legal proceedings.^22^, ^23^

## 5 BARRIERS TO CARE AND SUPPORT

The journey to seeking help is fraught with obstacles for survivors. Cultural stigma remains one of the most significant barriers, particularly in societies where discussing sexual violence is taboo. Survivors may internalize societal blame, believing they are responsible for the violence they endured, while marginalized individuals may face additional barriers related to stigma, identity‐based discrimination, and fear of negative responces.^2^, ^21^

Geographic, financial, and systemic disparities further impede access to post‐assault care, especially in rural or underserved areas. Survivors in such settings often face long travel distances, a lack of specialized sexual assault services, and inconsistent availability of trained healthcare professionals. Studies have shown that hospitals in rural settings frequently lack certified Sexual Assault Nurse Examiners (SANEs) and may be unable to provide timely, trauma‐informed care. Even when services exist on paper, staffing shortages and institutional constraints result in fragmented or delayed responses. The high cost of medical care, particularly in regions where survivors are uninsured or services are not subsidized, further discourages help‐seeking. These combined barriers contribute to survivors' reluctance to disclose or report incidents of sexual violence.^24^, ^25^, ^26^, ^27^

According to WHO, all victims of sexual violence are entitled to the protection of and respect for their human rights, including right to health, human dignity, non‐discrimination, self‐determination, information, privacy, confidentiality, as well as right to life, liberty, and security of the person, the right to be free from torture and inhuman, cruel, or degrading treatment.^14^

These challenges underscore the urgent need for stronger, more responsive health systems that can offer timely, respectful, and survivor‐centered care.

## 6 SURVIVOR‐CENTERED HEALTH SYSTEM RESPONSES AND GLOBAL MODELS

Over the past 15 years, FIGO has consistently worked to strengthen the role of the health sector in addressing violence against women. The FIGO Committee on Women Facing Crises strongly endorses that governments have a legal obligation not only to take all appropriate measures to prevent sexual violence but also to ensure that quality health services equipped to respond to sexual violence are available and accessible to all. Part of this obligation is to ensure that healthcare providers ensure an utmost respectful and sensitive attention to the survivor's needs.^14^

In its *Global Declaration on Violence Against Women* (2018), FIGO called on governments to strengthen health systems' capacity to prevent and respond to sexual violence, including training for healthcare professionals, survivor‐centered care, and integration of violence response into hospital policies and quality assurance programs.^28^ A coordinated, cross‐sectoral response—linking law enforcement, healthcare providers, and civil society organizations—is essential.^14^, ^23^, ^29^

As survivors who attend sexual assault centers are more willing to report, healthcare systems should have programs for the training of professionals as well as clinical protocols to intervene on behalf of patients who experience any type of violence. These protocols should feature prominently in hospital quality improvement programs.^14^, ^22^, ^30^, ^31^, ^32^ In support of this, FIGO's 2010 *Clinical Guidelines* offered foundational guidance for the care survivors, outlining standards for medical response, documentation, and survivor protection in healthcare settings.^33^

In support of these efforts, FIGO aligns with global calls, such as those made by WHO in its 2024 fact sheet and the *RESPECT Women* framework, to strengthen research and implement effective health sector responses to intimate partner and sexual violence.^34^, ^35^ In addition, through its Red Line initiative launched in 2022, FIGO partnered with the Royal College of Obstetricians and Gynaecologists (RCOG) to demand greater accountability for conflict‐related sexual violence and ensure that sexual and reproductive health services are prioritized even in humanitarian crises.^36^, ^37^

Several promising care models have been researched as a roadmap for comprehensively addressing the needs of survivors of sexual violence and could be implemented in other settings with contextual modifications, including the following:

1. Thuthuzela Care Centers (TCCs), South Africa: These hospital‐based centers provide integrated medical, legal, and psychosocial support, streamlining the process for survivors. Awareness campaigns associated with TCCs have increased survivor engagement and reporting rates.^38^
2. Sexual Assault Nurse Examiner (SANE) Programs, USA: Specialized training for nurses ensures trauma‐informed care and high‐quality forensic evidence collection, leading to increased survivor satisfaction and improved legal outcomes.^39^, ^40^
3. One‐Stop Crisis Centers (OSCCs), Malaysia, Pakistan: Located within hospitals, these centers combine medical, forensic, and psychological services, eliminating the need for survivors to navigate multiple agencies. Public education campaigns have significantly improved awareness and access.^41^, ^42^
4. The gender‐based violence and recovery center (GBVRC) model in Mombasa, Kenya: 24‐h emergency response services for sexual violence are combined with mental health support, paralegal services, and integrated cooperation with police, judiciary, local leaders, and the wider community.^43^

## 7 RECOMMENDATIONS FOR GOVERNMENTAL AUTHORITIES AND MINISTRIES

The FIGO Committee on Women Facing Crises continues to stress the need to draw a red line for sexual violence.^28^, ^33^, ^37^, ^44^, ^45^ Addressing sexual violence requires a comprehensive, multidisciplinary approach involving coordinated actions from the Ministries of Health, Justice, Social Affairs, and collaboration with non‐governmental organizations (NGOs). The FIGO Committee on Women Facing Crises emphasized targeted recommendations for each sector to address prevention, care, and justice effectively, incorporating strategies for psychological support and the critical role of NGOs in prevention, care, and advocacy (Table 1).^13^, ^14^, ^15^, ^18^, ^19^, ^22^, ^23^, ^31^, ^33^, ^46^, ^47^, ^48^, ^49^, ^50^, ^51^, ^52^, ^53^, ^54^, ^55^ Governments hold a crucial role in preventing sexual violence and supporting survivors. Governments have both legal and moral obligations to take appropriate measures and implement comprehensive strategies to ensure survivor protection, prevent sexual violence, and provide accessible, high‐quality care. Implementing these strategies requires sustained financial investment, workforce training, and robust monitoring systems to address resource constraints and ensure equitable access to survivor‐centered services. These responsibilities include:

1. Enacting comprehensive legal frameworks:
  - Criminalize all forms of sexual violence, including marital rape and child sexual abuse.
  - Develop survivor‐centered legal systems that protect survivors' dignity and ensure timely access to the justice system.
2. Allocating sustainable and targeted funding:
  - Invest in survivor‐centered facilities: one‐stop crisis centers, mobile outreach units, and community support programs.
  - Ensure funding education campaigns that challenge stigma, encourage reporting, and shift harmful norms.
  - Invest in trauma‐informed training programs for healthcare providers, to ensure compassionate, skilled, and culturally sensitive care.
3. Strengthening multisectoral collaboration:
  - Facilitate partnerships between law enforcement, healthcare providers, and NGOs to create coordinated and unified response systems.
  - Establish rapid response task forces that can act quickly in emergencies, including in conflict zones and marginalized areas.
4. Improving accessibility:
  - Expand critical services to rural and underserved areas through mobile clinics and telehealth initiatives.
  - Guarantee access to free or affordable health care and legal assistance, so no survivor is denied care because of cost or distance.
5. Promoting data‐driven accountability:
  - Implement national systems for data collection and monitoring to track the prevalence of sexual violence and the effectiveness of interventions.
  - Hold institutions accountable for gaps in service delivery and poor outcomes.
6. Protecting marginalized groups:
  - Ensure targeted protections and services for groups most at risk, including LGBTQ+ individuals, children, people with disabilities, and those in underserved or conflict‐affected regions.

Healthcare providers are uniquely positioned to identify sexual violence survivors as well as support and refer them to appropriate medical, psychological, and legal help. Training in trauma‐informed care enables providers to build trust, reduce stigma, and create a safe, respectful environment where survivors feel heard and empowered to seek care. Confidentiality, clear communication, and empathetic support are essential for fostering this sense of safety.^56^, ^57^, ^58^

**TABLE 1:** Targeted recommendations to address prevention, care, and justice.

The role and responsibilities of healthcare authorities/Ministry of Health	**Expand access to survivor‐centered healthcare services** Establish one‐stop crisis centers in hospitals and rural health facilities to provide integrated medical, forensic, and psychological care. Deploy mobile clinics and telehealth services in underserved areas to ensure geographical accessibility. **Standardize medical protocols** Develop and disseminate national clinical guidelines for the management of SV, including emergency contraception, STI prophylaxis, and psychological support. Ensure consistent forensic evidence collection practices to support legal proceedings. **Enhance healthcare provider training** Mandate trauma‐informed care training for all healthcare workers, focusing on sensitivity, confidentiality, and forensic documentation. Conduct regular refresher courses to keep providers updated on best practices. **Strengthen data collection and surveillance** Implement national systems to track the prevalence of SV and the utilization of healthcare services by survivors. Use data to identify service gaps and inform policy decisions. **Ensure confidentiality and respect** Develop clear policies to protect survivor privacy and eliminate mandatory reporting in cases where it deters survivors from seeking care. Prioritize culturally sensitive and inclusive practices to address the needs of marginalized groups. **Emergency preparedness** Ensure readiness to address SV during humanitarian crises through mobile clinics and rapid‐response teams. Develop contingency plans for pandemic‐related interruptions in survivor care.
Recommendations and responsibilities of the Ministry of Justice	**Reform legal frameworks** Ensure that legal definitions of SV align with international standards and encompass all forms of abuse. Abolish discriminatory laws that impede justice for survivors. **Streamline judicial processes** Establish fast‐track courts or specialized units to handle SV cases, minimizing delays and ensuring survivor‐friendly proceedings. Train judges, prosecutors, and law enforcement officials on trauma‐ informed approaches to handling SV cases. **Improve access to legal aid** Provide free legal assistance to survivors of SV, ensuring they have access to competent representation Partner with civil society organizations to create community‐based legal support networks. **Strengthen law enforcement capacity** Train police officers on handling SV cases with sensitivity and professionalism. Create dedicated gender‐based violence units within police departments to improve case outcomes and survivor trust. **Enforce accountability mechanisms** Monitor the performance of law enforcement and judicial officials in handling SV cases. Establish grievance redress mechanisms for survivors to report mishandling or bias.
Recommendations and responsibilities of Ministry of Social Affairs	**Provide comprehensive social support services** Develop shelters and safe houses for survivors, offering temporary housing, legal aid, and counseling. Establish survivor support hotlines staffed by trained counselors, with 24/7 availability. **Promote public awareness and education** Conduct nationwide campaigns to raise awareness about SV, its consequences, and available support services. Challenge societal norms that perpetuate stigma and victim‐blaming through community‐based education programs. **Empower survivors economically** Create vocational training programs to help survivors achieve financial independence. Provide grants or microloans to enable survivors to rebuild their lives. **Address vulnerable populations** Develop targeted programs for high‐risk groups, including children, LGBTQ+ individuals, and rural populations. Work with community leaders to create culturally sensitive interventions. **Foster peer support networks** Establish survivor‐led support groups to provide emotional and experiential guidance. Encourage survivors to share their stories through advocacy programs, helping to reduce stigma and empower others.
The role of NGOs	**Direct services to survivors** Provide immediate psychological support through hotlines, crisis centers, and mobile units. Offer long‐term counseling and therapy, often filling gaps left by governmental services. **Advocacy and awareness** Advocate for survivor rights and policy changes at local, national, and international levels. Conduct public education campaigns to challenge stigma and promote gender equity. **Capacity building** Train healthcare providers, law enforcement, and community leaders in trauma‐informed approaches and psychological first aid. Develop resources and tools, such as guides on supporting survivors, for use by stakeholders. **Community‐based support** Establish survivor‐led support groups that provide a safe space for sharing experiences and fostering mutual healing. Work with marginalized populations, including LGBTQ+ communities and rural residents, to address unique barriers to care. **Data collection and research** Conduct studies on the prevalence and psychological impact of SV to inform policy and service provision. Collaborate with governments to develop evidence‐based interventions.
Cross‐sector initiatives	**Develop integrated national action plans** Create comprehensive strategies that unify the efforts of health, justice, and social affairs ministries under a single framework. Include measurable goals and timelines to ensure accountability and progress. **Invest in research and innovation** Fund research on effective prevention and intervention strategies for SV. Support the development of innovative technologies, such as apps and helplines, to facilitate survivor reporting and access to services. **Engage men and boys** Promote gender equity by involving men and boys in community programs that challenge harmful norms and advocate for respectful relationships. Implement bystander intervention training to empower communities to prevent SV.

Abbreviations: NGO, non‐governmental organization; STI, sexually transmitted infection; SV, sexual violence.

If legal frameworks were reformed to align with international standards, survivors would gain clearer pathways to justice while discriminatory practices that deter reporting or hinder prosecution would be eliminated. Streamlining judicial processes through specialized units and trauma‐informed training would ensure that survivors are treated with dignity and cases are handled efficiently. Expanding access to legal aid, strengthening law enforcement capacity, and implementing accountability mechanisms all contribute to a justice system that survivors can trust and turn to. These measures collectively create a supportive environment that prioritizes survivor rights, enhances legal outcomes, and fosters systemic change in addressing sexual violence.^17^, ^59^, ^60^

Establishing comprehensive social support services, including shelters, hotlines, and counseling, would address immediate needs, while economic empowerment programs offer long‐term recovery pathways. Public awareness campaigns and culturally sensitive education are essential to dismantle stigma and challenge harmful societal norms. Peer support networks foster solidarity and resilience, offering survivors space for healing and opportunities to advocate for change. These measures collectively ensure holistic care and inclusion for diverse survivor populations.^61^, ^62^

NGOs are indispensable partners in addressing sexual violence by bridging gaps in services and advocacy often left unfulfilled by governments. Their direct support services address deeper psychological impacts. NGOs that are community‐based and focus on marginalized populations ensure inclusivity and accessibility, while data collection and research can contribute to informed policymaking and evidence‐based interventions. In settings where formal health services are limited, NGOs may also facilitate referral to medical facilities or support access to essential healthcare services for survivors. This multi‐faceted approach positions reproductive health NGOs as key drivers of both immediate care and long‐term solutions.^63^, ^64^

Cross‐sector initiatives are vital to effectively and sustainably address sexual violence. Integrated national action plans that bring together ministries, NGOs, and community help ensure coordinated strategies with real, measurable impact. By investing in research and innovation, countries can develop smarter prevention tools and expand access to supportive technologies and services.

In addition to strengthening response systems, primary prevention strategies are essential to reduce the incidence of sexual violence. Such prevention efforts should also address the social and cultural norms that shape attitudes toward gender and violence, particularly during childhood and adolescence when beliefs about relationships and power dynamics are formed. Educational initiatives that address harmful gender norms, promote gender equality, and foster respectful relationships from an early age can help shift social attitudes that tolerate or normalize violence. Community‐based programs involving educators, youth organizations, and local leaders play a critical role in promoting awareness, empowering young people, and encouraging collective responsibility for preventing sexual violence. Engaging men and boys in gender equity programs and bystander intervention training tackles the root causes of harmful behaviors, promoting cultural shifts toward respect and accountability.^65^ Together, these strengthen prevention, improve care, and build momentum for lasting cultural change.

Long‐term recovery and reintegration are essential components of survivor‐centered responses to sexual violence. Beyond immediate medical and legal assistance, survivors may require sustained psychological support, trauma‐informed counseling, social services, and economic empowerment programs to rebuild their lives. Community‐based support networks, peer support groups, and rehabilitation services can play an important role in fostering resilience and reducing long‐term social isolation. Strengthening systems that support survivors' long‐term recovery helps promote healing, restore dignity, and facilitate reintegration into families, communities, and society.^14^

## 8 CONCLUSION

Sexual violence is a pervasive global crisis with devastating consequences. Governmental authorities, alongside the ministries of health, justice, and social affairs, must take a proactive and collaborative approach to combat it. Addressing legal, social, and healthcare gaps through survivor‐centered policies and coordinated actions can help reduce its prevalence, ensure justice, and provide holistic care for those affected.

The recommendations presented in this paper offer a framework for sustainable, multidisciplinary, systemic change—one that ensures survivors receive the dignity, support, and justice they deserve.

Moreover, prioritizing psychological support, fostering survivor‐centered approaches, and leveraging the strengths of NGOs can help ensure that survivors are supported in their communities and have access to comprehensive, equitable care. Emphasis should also be placed on integrating innovative solutions, such as technology‐driven reporting platforms and data‐informed interventions, into national strategies.

Engaging men and boys as allies in prevention, promoting gender equity, and challenging harmful social norms are essential step toward addressing the root causes of sexual violence.

Inter‐sectoral activities must focus on long‐term, evidence‐based solutions that not only prevent violence, but also create environments where survivors are empowered and promote resilience. Through these combined efforts, societies can move toward a future where survivors are supported, justice is upheld, and the cycle of violence is broken.

## AUTHOR CONTRIBUTIONS

KJ, AR‐N, ME, RS, and LH contributed to the conceptualization of the consensus statement, the development of its recommendations, and critical review of the manuscript. KJ coordinated the manuscript preparation and led the drafting and revision process. AJ contributed to the literature synthesis, drafting, and revision of the manuscript. All authors reviewed and approved the final manuscript and agree to be accountable for the integrity of the work.

## FUNDING INFORMATION

No specific funding was received for this work.

## CONFLICT OF INTEREST STATEMENT

The authors have no conflicts of interest.

## Data Availability

Data sharing is not applicable to this article as no new data were created or analyzed in this study.
